# Expression of galectin-8 on human endometrium: Molecular and cellular aspects

**Published:** 2013-01

**Authors:** Hossein Nikzad, Hamed Haddad Kashani, Maryam Kabir-Salmani, Yoshihiro Akimoto, Mitsutoshi Iwashita

**Affiliations:** 1 *Anatomical Sciences Research Center, Faculty of Medicine, Kashan University of Medical Sciences, Kashan, Iran. *; 2 *Department of Molecular Genetics, National Institute of Genetic Engineering and Biotechnology (NIGEB), Tehran, Iran.*; 3 *Department of Anatomy, School of Medicine, Kyorin University, Mitaka, Tokyo, Japan. *; 4 *Department of Obstetrics and Gynecology, School of Medicine, Kyorin University, Mitaka, Tokyo, Japan.*

**Keywords:** *Endometrium*, *Galectin-8*, *Human*, *Western blot analysis*, *Immunohistochemistry*

## Abstract

**Background: **The up-regulation of galectin-3, galectin-9, and galectin-15 expression in the luminal and glandular epithelium was reported in preparation of the endometrium for embryo implantation at the midlutheal phase. However, no data was available regarding the expression and the distribution pattern of galectin-8 in the human endometrium during a regular menstrual cycle.

**Objective:** The current study designed to investigate the expression and the distribution pattern of galectin-8, a beta-galactoside-binding lectin in the human endometrium during both proliferative and luteal phases of a regular menstrual cycle.

**Materials and Methods: **Endometrial biopsies were obtained from the anterior wall of the uterine cavity of 16 women (proliferative phase: n=4, lutheal phase: n=12). All female patients with mean age of 37.5 years were fertile (range 25-45)**.** Each biopsy was divided into three pieces; one piece was fixed in formaldehyde for light microscopy and immunohistochemistry. The second portion fixed in glutaraldehyde for scanning electron microscopy and the third portion was prepared for western blot analysis.

**Results:** Data of immunoblotting revealed a molecular weight of 34 kD band with high intensity in the lutheal phase samples. The immunohistochemistry staining demonstrated that galectin-8 expressed at a very low concentration during the proliferative phase, but showed a high expression throughout the lutheal phase. The expression of galectin-8 observed in luminal surface epithelium, glandular epithelium and stroma.

**Conclusion:** The up-regulation of the expression of galectin-8 during lutheal phase may suggest galectin-8 as one of the potential molecular marker of the endometrial receptivity. These data propose that galectin-8 may play an important role during the initial events of human embryo implantation.

## Introduction

Preparation of the endometrium for implantation depends on adequate hormonal stimulation and the presence of appropriate mediators at the endometrial-blastocyst interface (1). Implantation window is characterized by remarkable ultrastructural changes in the apical surface of luminal uterine epithelium, named pinopod as well as the up-regulation of several endometrial growth factors, cytokines and adhesion molecules in the luminal epithelium (2-5). 

Integrins, lectins, and cadherins family of adhesion molecules appeared to contribute in the attachment of blastocyst to endometrial epithelium (6). Lectins are proteins that bind to the specific carbohydrate structures and can thus recognize particular glyco-conjugates among the vast array expressed in animal tissues. In most animals, lectins are classified into four distinct families: C-type lectins (including the selectins); P-type lectins; pentraxins; and galectins, formerly known as S-type or S-Lac lectins (7). 

To date, 15 galectins have been identified. The galectins are small soluble proteins, defined by a carbohydrate recognition domain (CRD) with affinity for β-galactosides and a conserved sequence motif (8, 9). Galectin-8, a member of galectin family with a 34 KDa weight, was initially cloned from a rat liver cDNA library and is one of the most widely expressed galectins in human normal tissues and cancer tissues (10-12).

Galectin-8 reported to play a role in the regulation of cell growth, cell adhesion, migration, apoptosis, inflammation, and immunomodulation, all of which are important for endometrial function during embryo implantation (13-15). Levy *et al* demonstrated that galectin-8 was involved in mediating cell-cell interactions through its binding to a subset of integrin family including, α3β1, and α6β1, but not α4β1 integrins (16). Furthermore, integrins α1β1, α1β4, and αvβ3 reported to be expressed in uterine epithelium during the frame of implantation window (17, 18). 

Several previous studies have shown up-regulation of galectin-3, galectin-9 and galectin-15 in the luminal and glandular epithelium at the mid-lutheal phase (19-23). However, the expression and the distribution pattern as well as the biological function of galectin-8 in the human endometrium were unknown. Thus, this study was designed to investigate the expression and the distribution pattern of galectin-8 in the human endometrium.

## Materials and methods

***Endometrial specimens***

As a cross-sectional study, endometrial biopsies were obtained from the anterior wall of the uterine cavity of 16 women. The Ethics Committee of Kyorin Medical University approved the design of the study and informed consent was obtained from all participating women. 

All women were fertile with regular menstrual periods (25-35 days). The mean age was 37.5 years (range 25-45) and none of them had used steroidal contraceptive or an intrauterine device for at least 3 month before sampling. Each biopsy sample was divided into three pieces: one was fixed with 10% neutrally buffered formaldehyde for light microscopy and immunohistochemistry. The second portion was fixed with 2.5% glutaraldehyde in 0.1 M phosphate buffer (pH 7.4) for scanning electron microscopy and the third portion was prepared for Western blot analysis. For endometrial dating, endometrium samples were dated according to the histopathological criteria of Noyes and expression of pinopodes into four equal experimental groups: proliferative (days 6-14), early luteal (days 15-19), mid-luteal (days 20-24) and late luteal (days 25-28) (24).

**Scanning electron microscopy**

Scanning electron microscopy was used in this study to confirm the presence of pinopodes in the endometrial samples. For scanning electron microscopy preparation, endometrial tissues were fixed for at least 24 hr in 2.5% glutaraldehyde in 0.1 M phosphate buffer (pH 7.4) and post fixed for 1hr in 1% Osmium Tetroxide in 0.1 M phosphate buffer (pH 7.4). 

The samples were dehydrated in a graded series of ethanol (50%, 70%, 90%, 99.5% and 100%), critical-point-dried with carbon dioxide by using a freeze drying device (JFD–300, JEOL, Tokyo, Japan), mounted and coated with gold in a sputter coater (JFC-1300 Auto Fine Coater, JEOL, Tokyo, Japan). Finally, the samples were examined by using a scanning electron microscope (JSM- 5600 LV SEM, JEOL, Tokyo, Japan).

**Immunostaining for light microscopy**

To study the expression pattern of galectin-8 in human endometrium, immunehistochemical staining was performed. For standard immunohistochemical technique, paraffin sections (4 µm) were dewaxed in xylen and rehydrated in decreasing concentrations of ethanol and, finally distilled water. Endogenous peroxidase was blocked by 0.3% hydrogen peroxide in methanol for 10 min and nonspecific antibody binding was blocked by incubation in 5% BSA in PBS for 30 min. 

After this treatment, the sections were washed three times (5 min each) with PBS and incubated with goat polyclonal galectin-8 antibody (Santa Cruz Biotechnology, Inc). The antibody was applied at a concentration of 1.5g/ml over night at 4^o^C. After washing three times (5 min each) with PBS, the sections were incubated with horseradish peroxidase (HRP)-conjugated donkey anti goat antibody (1:250 diluted) for 1 hr at room temperature and washed again three times with PBS. 

Peroxidase activity was revealed by incubation with the 3, 3’-diaminobenzidine tetrahydrochloride (DAB)-H_2_O_2 _reaction (0.5 mg/ ml PBS) containing 0.005% H_2_O_2_ for 10 min at room temperature and then washed with distilled water. Finally, slides were counterstained with hematoxylin and mounted. For negative controls, normal rabbit serum IgG was used substituting for the primary antibody in the staining protocol. 

**Western blot analysis**

At biochemical level, to study the expression of galectin-8 in human endometrium, Western blotting was performed. Endometrium samples were sonicated in PBS (pH 7.2) containing 1% protease inhibitor. Then insoluble materials were removed by centrifugation at 20,000 xg for 15 min and the amount of protein in the supernatants was determined by Bradford method. 

Laemmli sample buffer was added to 10µg/mL of lysates and proteins were boiled for 10 min, electophoresed in 12.5% SDS-PAGE and transferred to polyvinylidene difluoride (PVDF) membranes. The membranes were blocked with TBS-T solution containing 3% BSA for 4h at room temperature, then incubated with goat polyclonal anti-galecin-8 diluted with blocking buffer (1:1000) at 4^o^C for overnight. Following several washes with washing buffer [Tris buffer containing 0.5% (v/v) Tween-20], immunoreactive proteins were identified by incubation with horseradish peroxidase-conjugated donkey anti-goat IgG diluted with blocking buffer (1:10000) for 1 h at room temperature. 

Primary and secondary antibodies were purchased from Santa Cruz Biotechnology, Inc. (Santa Cruz, CA, USA). After several washes with washing buffer, membrane was visualized with enhanced chemiluminescence (ECL) reagents (Pierce USA) and exposed to Kodak X-AR film (Eastman Kodak Co., Rochester, NY, USA) for 1-5 min at room temperature. This experiment was performed in three samples of each group. 

## Results

**Figure 1:**
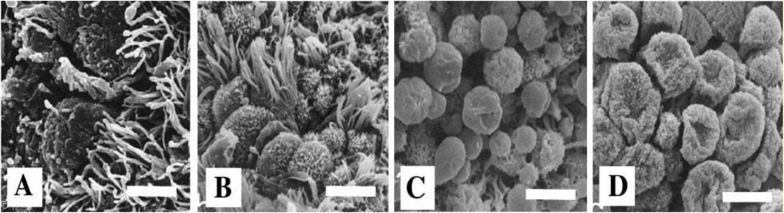
Scanning electron microscopy photomicrographs of luminal surface of human endometrial biopsies were taken from proliferative (A), early (B), mid- (C) and late luteal (D) phases of normal menstrual cycle to identify developmental stage of pinopodes. Notice that there are not any pinopodes in proliferative phase, few isolated pinopodes are detectable in specimens from early luteal phase, numerous developed pinopodes are detectable in the mid-luteal phase, and regressive pinopodes are dominant in late luteal phase specimens. Scale bar = 5 µm.

Images from scanning electron microscopy demonstrated that endometrial epithelium in secretory phase shows two different types of cells: ciliated and non-ciliated cells, that latter cover the majority of luminal surface (Figure 1). Membrane projections on the apical pole of non-ciliated cells appear as fine microvilli and dome-like projections defined as progressing, developed and regressing pinopodes (2). 

Comparing images from early, mid luteal and late luteal phase specimens revealed that in the early luteal phase (dating 15-19) progressing pinopodes, in mid luteal phase (dating 20-24) fully developed pinopedes and in late luteal phase (dating 25-28) regressing pinopodes are dominant (Figure 1 b, c, d). In images of proliferative phase specimens, there is not any pinopodes on the epithelium surface (Figure 1 a). 

**Figure 2:**
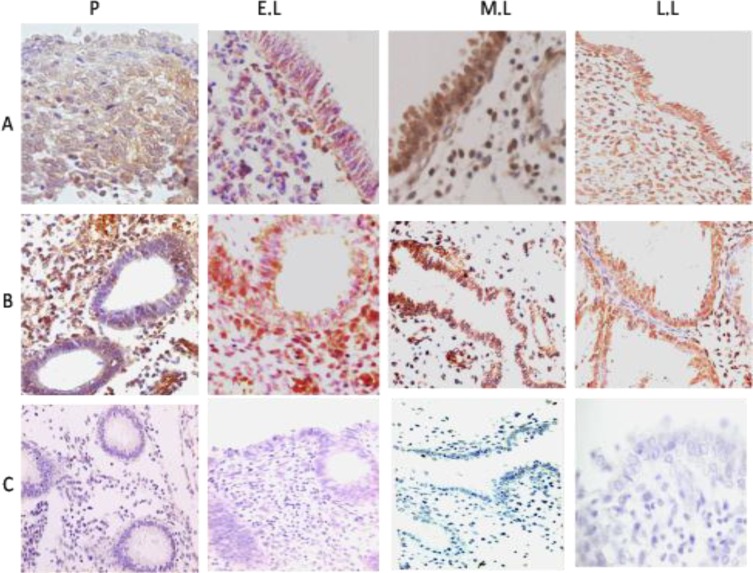
immunostaining for galectin-8 expression in the biopsies from proliferative (P), early (E.L), mid- (M.L) and late luteal (L.L) phases of normal menstrual cycle in human endometrium. Immunoreactive galectin-8 is seen in the uterine luminal surface epithelium (row A), glandular epithelium and stroma (row B), which is more intense in the luteal phase samples. Row C shows the negative control with normal goat-IgG (Magnification ×400).

**Figure 3:**
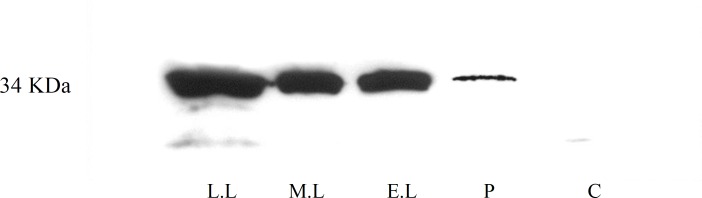
Western blotting for galectin-8 demonstrated a band of protein mass with molecular weight of 34 kDa in endometrium biopsies from late luteal (L.L), mid-luteal (M.L), early luteal (E.L), and proliferative (P) phases. Using the negative control antibody (C) resulted in no band, which approved the specificity of the experiment

Immunohistochemistry staining using DAB method demonstrated that galectin-8 expressed at very low concentration during the prolifrative phase, but showed a strong expression throughout lutheal phase (Figure 2). The expression of galectin-8 was exhibited in luminal surface epithelium, glandular epithelium and stroma. Substitution of normal goat IgG for galectin-8 primary antibody resulted in no staining as shown in figure 2. Results obtained from Western blotting experiments indicate the existence of a protein mass of 34 kDa corresponding to galectin-8 molecule. Using the negative control antibody resulted in no band, which approved the specificity of the experiment (Figure 3).

## Discussion

The endometrium is hormonally regulated throughout the menstrual cycle, and exhibits a short period of receptivity, known as the ‘implantation window’ (25). In humans, the endometrium becomes receptive to blastocyst implantation 6-8 days after ovulation and remains receptive for 4 days (26). During the implantation window, an invasive embryo must attach itself to the luminal endometrium under conditions of shear stress. This process is triggered by a complex series of events that are divided into the three overlapping steps in human model, termed as apposition, attachment, and invasion (27). The process of embryo implantation in humans bears some similarity to leukocyte transmigration across the blood vessel wall. Accordingly, some studies have shown that the initial interactions between leukocytes and vascular endothelial cell surface are mediated by lectins (28). 

There are parallels between leukocyte extravasation from the vasculature and the attachment of embryo to the uterine wall considering both types of adhesion occur under a shear flow and are mediated by integrin activation. Consistently, galectins bind to cell adhesion molecues and are potential candidates to support the binding between endometrial luminal epithelium and blastocyst during attachment phase of embryo implantation. 

On the basis of these facts and considering that the expression of galectin-3, galectin-9, and galectin-15 was up-regulated in the luminal and glandular epithelium during the implantation window (19-23); and regarding that no data was available concerning the expression of galectin-8 in the human endometrium, this study was designed to investigate the possible expression and modification of this lectin during the menstrual cycle.

The results of immunohistochemistry showed that galectin-8 is highly expressed at the luminal surface epithelium, glandular epithelium, and stroma in the lutheal phase. In the previous work, we reported co-expression of galectin-3 with αvβ3 integrin on luminal epithelium surface of human endometrium in the mid-luteal phase (19). Expression of galectin-3 increases significantly during the secretory phase of the menstrual cycle. It can modulate cell adhesion by binding to ligands including laminin, fibronectin and integrins after its secretion from epithelial cells (18). 

Galectin-9 has been identified in mid- and late-secretory and decidual phases in human endometrium, with expression in glandular and luminal epithelial (20). Expression of galectin-9 on uterodomes suggests that galectin-9 may play a role during the initial events of human embryo implantation (21). Galectin 15 is regulated by progesterone and is secreted from the endometrial luminal epithelium in sheep, where it has a prospective role in trophectoderm attachment (22). It is proposed that immobilized galectin-8 may function as a matrix ligand similar to fibronectin in promoting the cell adhesion by binding and clustering integrin receptors. Furthermore, it has been postulated that galectin-8 as a soluble ligand can form a complex with integrins that negatively regulates cell adhesion (16). Consistently, Hadari *et al *demonstrated that galectin-8 inhibits adhesion of human carcinoma cells to plates coated with integrin ligands, and induces cell death by apoptosis (15). 

Nishi *et al* reported that galectin-8 modulates neutrophil functions related to transendothelial migration and microbial killing via interaction with integrin-αM (29). Moreover, it has been reported that in a colony formation assay, the transfection of galectin-8 cDNA into cells significantly reduced colony growth. They reported that major binding protein was integrin-α3β1, although galectin-8 could interact with other members of the integrin family, like integrin-α6β1 as well. In the mentioned study, minimum interaction was observed between galectin-8 and integrin-α4 and integrin-β3 (30). Furthermore, Cárcamo *et al* reported that galectin-8 interacted with specific β1 integrins (integrin-α1β1, integrin-α3β1and α5β1) and induced extensive cell spreading in T-cells (31). 

Therefore, it can be concluded that binding of galectin-8 may modulates integrin interactions with the extracellular matrix and thus regulates cell adhesion and cell survival. Moreover, it may modulate a wide range of immune processes driven by T-cells that eventually become altered in autoimmune disorders. According to the above reports, galectin-8 play a role in the regulation of cell growth, cell adhesion, migration, apoptosis, inflammation, and immunomodulation (13-15), all of which are important for endometrial function, as well as implantation.

## Conclusion

In conclusion, the presence of galectin-8 on the luminal epithelium surface of the human endometrium especially its up-regulation at the midlutheal phase may provide an explanation for its role as a modulator of attachment in the implantation site.
